# Survival and Impact on Microbial Diversity of *Lacticaseibacillus paracasei* DG in a Simulation of Human Intestinal Microbial Ecosystem

**DOI:** 10.3390/nu17182952

**Published:** 2025-09-13

**Authors:** Cindy Duysburgh, Walter Fiore, Massimo Marzorati

**Affiliations:** 1 ProDigest, Technologiepark-Zwijnaarde, 9052 Ghent, Belgium; 2 Alfasigma S.p.A., Kilometro Rosso, Via Stezzano, 87, 24126 Bergamo, Italy; 3 Center of Microbial Ecology and Technology (CMET), Ghent University, Frieda Saeysstraat, 9052 Ghent, Belgium

**Keywords:** *Lacticaseibacillus paracasei* DG, probiotics, SHIME, survival, metabolic activity, colonic microbial diversity

## Abstract

**Background/Objectives:** The probiotic *Lacticaseibacillus paracasei* DG (LpDG) has shown promising results for various gastrointestinal diseases. This study evaluated the survival, metabolic activity, and impact on colonic microbiota of LpDG in an in vitro gastrointestinal tract simulation. **Methods:** Encapsulated LpDG was tested under simulated fed, fasted, and shortened fasted conditions compared with a blank control in a modified Simulator of the Human Intestinal Microbial Ecosystem (SHIME^®^) reactor. Capsule integrity, and cell culturability and viability were assessed at the end of each digestion phase. Metabolic activity (pH, total gas production, and concentrations of short-chain fatty acids, lactate, and ammonium) was assessed after a 24 h colonic incubation with a faecal inoculum. The impact of LpDG on the colonic microbial community was analysed by quantitative polymerase chain reaction and shallow shotgun sequencing. **Results:** The capsule was completely degraded at the end of the jejunum under all conditions. A low pH had a minimal impact on LpDG culturability and viability. Compared with blank control, LpDG remained metabolically active in the microbial community following a 24 h colonic incubation (LpDG [0–24 h] vs. blank control [0–24 h]: ΔpH, decreased [0.29–0.38 vs. 0.12–0.34]; Δlactic acid, decreased [1.52–1.69 mM vs. 0.13–0.21 mM]; and Δbutyrate, increased [7.49–10.52 mM vs. 5.19–7.76 mM]). Under fed conditions, treatment with LpDG compared with blank control significantly decreased levels of *Escherichia coli* and *Blautia wexlerae* and increased Clostridiaceae, Eubacteriaceae, and Lachnospiraceae. **Conclusions:** LpDG remains viable and metabolically active in the gastrointestinal tract, positively affecting intestinal microbiota and metabolite production.

## 1. Introduction

Probiotic treatments can help to improve lower gastrointestinal (GI) symptoms, as shown by Delphi consensus statements [1,2]. The bacteria in the extremely diverse *Lactobacillus* genus (referring to all *Lactobacillus* bacilli before reclassification in 2020) are Gram-positive, anaerobic, fermentative, and non-spore-forming bacilli capable of producing lactic acid in the GI and genitourinary tracts [3,4]. In 2020, the genus was reclassified into 26 genera based on phylogenetic parameters, and physiological and ecological criteria. The reclassified genus *Lacticaseibacillus* refers to the heterogeneous casei-group of homofermentative, non-motile, oxidase-negative lactobacilli [3]. The strain *Lacticaseibacillus paracasei (L. paracasei)* DG^®^ (DG I-1572, DSM 34154; L. casei DG^®^), previously known as *Lactobacillus paracasei* [3], is a probiotic bacterium that has been shown to survive the passage through the upper GI tract in both healthy adults and children [5,6]. Treatment using *L. paracasei* DG as a single-strain probiotic has shown promising results in the treatment of symptomatic uncomplicated diverticular disease (SUDD) [7,8]. It has also been shown to reduce inflammation in the colonic mucosa of patients with mildly active ulcerative colitis, to help to reduce side effects during treatment for *Helicobacter pylori*, and to improve symptoms of irritable bowel syndrome (IBS) for a small subset of patients [9,10,11,12].

Clinical trials can only provide information on input and output of the probiotic, and the mechanism by which *L. paracasei* DG survives the passage through the gut has not been well characterized. To confer benefit, probiotics must be viable and metabolically active at their site of activity (distal ileum and colon). Probiotics must pass several safety and functionality criteria, including being acid resistant, bile salt resistant, non-pathogenic, and tolerant of harsh processing conditions, as well as needing to produce lactic acid [13,14]. Once delivered to the distal ileum and colon, probiotics can directly or indirectly modulate the host’s colonic fermentation process through a host–microbiota interaction [13,15].

Intake of *L. paracasei* DG results in increased levels of Proteobacteria and Clostridiales genus *Coprococcus* and decreased levels of Clostridiales genus *Blautia* in healthy human faecal samples; this probiotic effect has been shown to depend on the initial faecal butyrate concentration [16]. Sampling the microbiota at different stages of human colonic passage is not possible [17], and, despite significant overlap among species, murine gut flora are not representative of those in humans [18]. Specifically, the most distinct differences between mice and humans are in the ratio of two major phyla: Firmicutes and Bacteroidetes [18].

In vitro models simulating the physiological conditions of the complete upper GI tract allow for sampling of the intestinal microbial community from each region of the tract, without the need for invasive methods, to provide in-depth mechanistic insights into the mode of action of probiotics [17]. The Simulator of the Human Intestinal Microbial Ecosystem (SHIME^®^) is an in vitro dynamic model of the whole human GI tract, consisting of five reactors simulating the stomach, small intestine, and ascending, transverse, and descending colon, which is inoculated with the faecal microbiota of selected donors [17,19].

This study employed a modified version of the SHIME^®^ reactor, simulating the stomach, small intestine, and proximal (ascending and transverse) colon, to evaluate the survival, culturability, and impact on colonic microbial diversity of *L. paracasei* DG under fed, fasted, and shortened fasted conditions. The study, therefore, addresses a critical gap in our understanding of the survival and metabolic activity of *L. paracasei* DG in the human GI tract.

## 2. Materials and Methods

### 2.1. Composition of Capsule Systems

*L. paracasei* DG was encapsulated in a vegetal capsule (hydroxypropyl methylcellulose; colouring, calcium carbonate E170) with anti-agglomerant agents (silicon dioxide and magnesium salts of fatty acids) at a concentration of 24 billion colony-forming units (CFU)/capsule (as of the end of shelf life). Capsules were mounted in a capsule sinker (Prosense BV, Oosterhout, The Netherlands), and one capsule was dosed per reactor. Incubation with encapsulated *L. paracasei* DG was compared with a blank control incubation. All experiments were performed in triplicate.

### 2.2. Modified In Vitro SHIME^®^ Experiments

**Figure 1:**
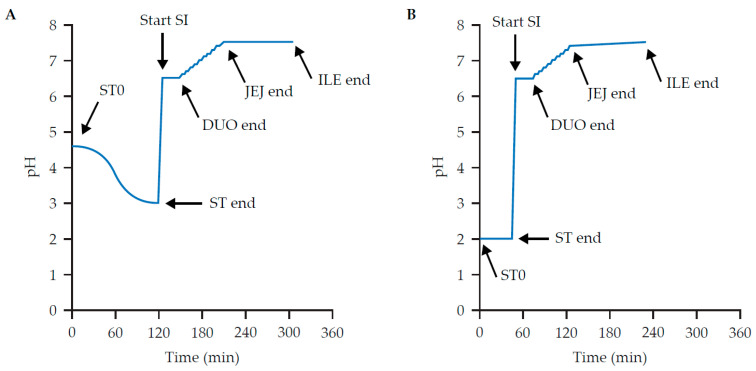
pH profiles for fed (**A**) and fasted (**B**) conditions during the stomach and small intestinal phases.

A modified SHIME^®^ reactor, adapted from the protocol by Molly et al. [19], was used to represent the physiological conditions of the stomach, small intestine, and proximal colon in the same reactor over time (Supplementary Figure S1). The protocol has been described previously [20]. In brief, a single pH- and temperature-controlled reactor was used to simulate first the stomach and then the small intestinal environments under fed, fasted, or shortened duration of fasted conditions (hereafter referred to as ‘shortened fasted’ condition). Incubation phases consisted of the start of the stomach phase (ST0), the end of the stomach phase (ST end), the duodenal phase (DUO), the jejunum phase (JEJ), and the ileal phase (ILE). The temperature was maintained at 37 °C throughout all experiments. Representative conditions for fed and fasted simulations were modified from the European Cooperation in Science and Technology Action InfoGest consensus methods [21] and a protocol by Riethorst et al. [22], respectively. pH profiles and incubation times were optimized and controlled for the fed (Figure 1A) and fasted (Figure 1B) conditions, consistent with previous protocols and the reported in vivo variability [22,23].

### 2.3. Simulated Stomach and Small Intestinal Digestions

For the fed condition, stomach digestion was simulated by the incubation of simulated gastric fluid (SHIME^®^ nutritional media [PDNM001B; ProDigest, Gent, Belgium] containing arabinogalactan, pectin, xylan, starch, glucose, yeast extract, peptone, mucin, and L-cysteine HCl) with pepsin and phosphatidylcholine.

Salt levels were in accordance with those in the European Cooperation in Science and Technology Action InfoGest consensus methods [21]. Samples were incubated for 2 h at 37 °C with continuous mixing. To mimic the fed condition, the pH profile was decreased from 4.6 to 3.0 (Figure 1A).

For the fed condition, the simulated gastric fluid was combined with raw animal pancreatic extract containing all relevant enzymes and 10 mM bovine extract, as previously described [20,22]. To simulate intestinal digestion, the pH was increased from 3.0 to 6.5 and kept constant at 6.5 over a 27 min period (mimicking the DUO) (Figure 1A). This phase was followed by a stepwise pH increase to 7.5 over the course of 63 min, representing the JEJ. Finally, the pH remained at 7.5 for 90 min (ILE).

The tests were conducted similarly for the fasted and shortened fasted conditions, with the following modifications [22]. For the fasted ST, the pH was maintained at 2.0 for 45 min (compared with the decrease of 4.6 to 3.0 in the fed condition). Small intestinal digestion times were identical to those used for the fed condition. Fourfold lower pepsin and fourfold lower phosphatidylcholine were added to the background medium for the fasted condition compared with those for the fed condition, and the background medium contained only salts and mucins (no addition of arabinogalactan, pectin, xylan, starch, glucose, yeast extract, peptone, or L-cysteine HCl). During the intestinal phase, pancreatic enzyme levels and bile salts were fivefold and threefold lower, respectively, than in the fed condition.

The ‘shortened fasted’ condition was included to represent an intermediate state between fed and fasted states, and to account for the variability in gastric emptying times and luminal conditions between individuals and circumstances. For the shortened fasted condition, the incubation time for the ST was 10 min (compared with 45 min for the fasted condition). Modifications to the pH and concentrations of pepsin and phosphatidylcholine were the same as for the fasted condition. The small intestinal digestion times were shortened from the fasted condition to 13.5 min (DUO), 31.5 min (JEJ), and 45 min (ILE).

Sampling and visual scoring were conducted at baseline, at the end of the ST phase, and after each small intestinal phase.

### 2.4. Visual Assessment of Capsule Integrity

Visual assessment of the capsule integrity was conducted before digestion (unprocessed product) and at the end of each simulated phase of the upper GI tract (ST end, DUO end, JEJ end, and ILE end). Capsule integrity was classified as follows: (1) intact capsule, no product released; (2) capsule damaged but almost all product is still in the capsule; (3) capsule damaged and all product released; and (4) capsule destroyed and all product released.

### 2.5. Quantification of CFU

*L. paracasei* DG CFU were assessed before product digestion and at the end of the ST and small intestine phases by 10-fold serial dilutions in anaerobic phosphate-buffered saline on de Man, Rogosa, and Sharpe agar plates. Plates were subsequently incubated aerobically at 37 °C for at least 24 h.

### 2.6. Quantification of Viable and Non-Viable Bacterial Cells by Flow Cytometry

Samples collected at the start and at the ends of the ST, DUO, JEJ, and ILE phases were assessed by SYTO 24 and propidium iodide staining. Samples were analysed using a BD FACSVerse (BD Biosciences, Franklin Lake, NJ, USA).

### 2.7. Short-Term Colonic Incubation Experiments

Colonic incubations were performed for encapsulated *L. paracasei* DG with a representative bacterial inoculum as a simulation for the proximal large intestine. A fresh faecal sample was collected from a single healthy human donor and used as the bacterial inoculum source. The donor had an omnivorous diet and did not use antibiotics in the 4 months before the study start. At the start of the short-term colonic incubation period, SHIME^®^ nutritional medium (PD01), containing basal nutrients that are present in the colon, was mixed with freshly prepared faecal inoculum and added to the simulation medium derived from the upper GI tract incubations. Incubations were performed anaerobically for 48 h with shaking (90 rpm). Simulation medium from a blank upper GI tract control incubation was used as the negative control. Sampling was conducted at baseline and at 24 h.

### 2.8. Microbial Metabolic Activity

Microbial metabolic activity was assessed at 0 h and 24 h of incubation as previously described [20]. *L. paracasei* DG-supplemented colonic incubations were compared with blank control incubations for changes in pH, total gas production, and short-chain fatty acid (SCFA; specifically acetate, propionate, and butyrate), branch-chain fatty acids (BCFA), lactate, and ammonium concentrations.

### 2.9. Microbial Community Composition

*L. paracasei* DG counts were assessed by quantitative polymerase chain reaction (PCR) after 0 h and 24 h of incubation in the SHIME^®^ colonic environment (Supplementary Figure S2). The final primer concentration for *L. paracasei* DG was 0.7 µM. The conditions used were an annealing temperature of 60 °C, an annealing time of 30 s, an extension temperature of 72 °C, and an extension time of 30 s.

Shallow shotgun sequencing was performed to evaluate the modulation of the microbial composition upon exposure to *L. paracasei* DG compared with blank control. Samples were taken after 0 h and 24 h of incubation in the SHIME^®^ colonic environment. Briefly, after DNA extraction, DNA libraries were prepared using the Illumina Nextera XT library preparation kit. Library quantity was assessed with Qubit (ThermoFisher, Waltham, MA, USA), after which libraries were sequenced on an Illumina HiSeq platform 2 × 150 bp. Overall, 3 million reads were generated from the microbial community genomes. Unassembled sequencing reads were analysed for multi-kingdom microbiome analysis. Profiling of antibiotic resistance and virulence genes and quantification of each assessed organism’s relative abundance, using curated genome databases and a datamining algorithm that disambiguates metagenomic sequence reads into discrete microorganisms, were also performed (CosmosID Inc., Germantown, MD, USA) [24,25].

### 2.10. Data Analysis

For the endpoints of the upper GI tract simulation, two-tailed paired Student’s *t*-tests were used for the comparison of survival at a specific time point to the preceding time point. For the colonic endpoints, a two-tailed homoscedastic *t*-test was used for the comparison of the different treatments to the blank control. Differences were considered statistically significant if *p* < 0.05. The normality of the data was confirmed based on historical data. The analysis was conducted using Microsoft Excel.

## 3. Results

### 3.1. Capsule Integrity and Survival of L. paracasei DG Under Fed, Fasted, and Shortened Fasted Conditions

**Figure 2:**
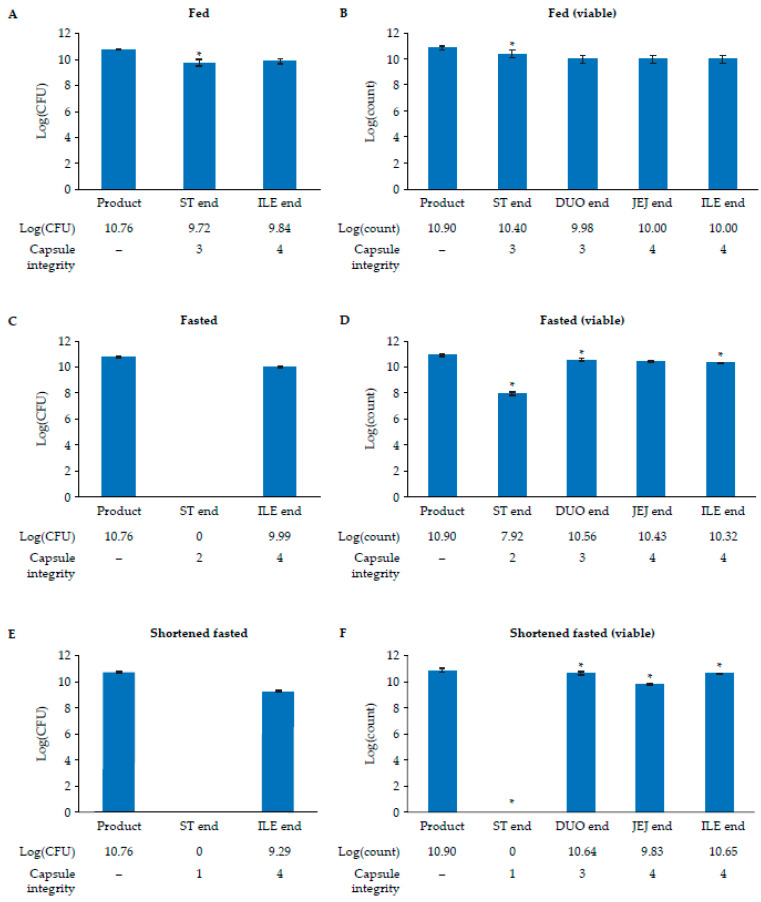
Capsule integrity and survival of *L. paracasei* DG transiting through the SHIME^®^ GI tract under fed, fasted, and shortened fasted conditions. Mean log(CFU) (SD) (*n* = 3) by spread plating (**A**,**C**,**E**) and mean log(count) (SD) (*n* = 3) by flow cytometry (**B**,**D**,**F**) of *L. paracasei* DG during passage through the upper GI tract under fed (top), fasted (middle), and shortened fasted (bottom) conditions. Data are representative of samples taken of the product before processing and samples taken at the end of the ST, DUO, JEJ, and ILE phases. Spread plating for 24 h on de Man, Rogosa, and Sharpe agar plates assessed the cell culturability of released *L. paracasei* DG following incubation at different stages of digestion. The limit of quantification was log(CFU) < 3.5. Viability was determined by flow cytometric staining of *L. paracasei* collected with SYTO and propidium iodide. * Represents statistical significance between the current and preceding time point (*p* < 0.05). The visual scores of the capsules are indicated in the table below the bars: (1) intact capsule, no product released; (2) capsule damaged but almost all product is still in the capsule; (3) capsule damaged and all product released; and (4) capsule destroyed and all product released.

The durability of the capsule and subsequent survival of encapsulated *L. paracasei* DG as the capsule transited through the GI tract were assessed in the fed, fasted, and shortened fasted conditions (Figure 2). Under fed conditions, the capsule was partially degraded at ST end (visual score: 3) and fully degraded by JEJ end (visual score: 4) (Figure 2A). The *L. paracasei* DG released at ST end was culturable but experienced a logarithmic reduction by approximately one unit in total culturability compared with the unprocessed product (Figure 2A). Culturability partially recovered to a log(CFU) of 9.84 by ILE end. Viability, assessed by flow cytometric analysis of released *L. paracasei* DG, was minimally affected: the initial log(count) of 10.90 in the unprocessed product decreased to 10.40 by ST end, 9.98 at DUO end, and 10.00 at JEJ end (Figure 2B).

Under fasted conditions, the capsule was damaged at ST end (visual score: 2) and was fully degraded by ILE end (visual score: 4; Figure 2C). The cells were not culturable at ST end, remaining below the limit of quantification (log[CFU] < 3.5). Compared with the unprocessed product, the number of viable cells was reduced by three logarithmic units at ST end (Figure 2D). During the product’s transit through the remaining GI phases, the capsule was completely degraded, and the culturability and viability of the *L. paracasei* DG cells rebounded from ST end to a log(CFU) of 9.99 and log(count) of 10.32, respectively, at ILE end.

Under shortened fasted conditions, the capsule was intact at ST end and was fully digested by ILE end (visual score: 4; Figure 2E). The log(CFU) of *L. paracasei* DG remained below the limit of quantification at ST end as the probiotic bacterium was not released; however, by ILE end, the culturability of *L. paracasei* DG increased from undetectable at ST end to a log(CFU) of 9.29, more than one logarithmic unit lower than that of the unprocessed product. Moreover, viability and counts remained high upon release of *L. paracasei* DG from the capsules in the GI tract (ILE end, log[count] = 10.65), at levels comparable to the unprocessed product (log[count] = 10.90) (Figure 2F).

### 3.2. Microbial Fermentation of Human Faecal Inoculum Following the Administration of L. paracasei DG

**Figure 3:**
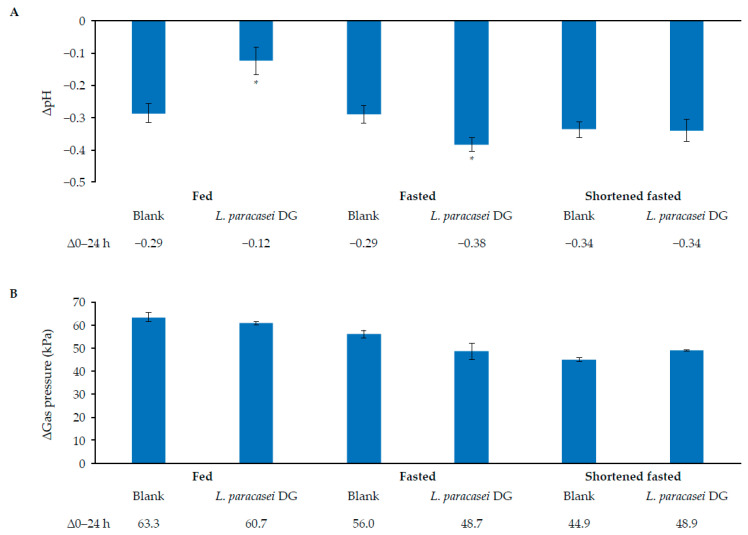
Overall microbial metabolic activity in short colonic incubations. Change (mean [SD]) in pH (**A**) or gas pressure (kPa) (**B**) upon colonic fermentation during 0–24 h in the presence of *L. paracasei* DG compared with blank control under fed, fasted, and shortened fasted conditions. *n* = 3 technical replicates. * Represents statistically significant differences compared with blank control for the respective condition (*p* < 0.05).

In a blank colonic control incubation with healthy human faecal inoculum for 24 h, the pH changed from baseline by between –0.34 and –0.29 across the fed, fasted, and shortened fasted conditions (Figure 3A). The addition of the *L. paracasei* DG capsules significantly reduced the pH under fasted conditions after 24 h compared with the blank incubation (–0.38 vs. –0.29; *p* < 0.05). However, shortened fasted conditions led to a similar decrease of pH, and fed conditions led to a smaller decrease of pH compared with the blank control incubation. Blank control incubation for 24 h also increased the rate of gas production by approximately 45–63 kPa under fed, fasted, and shortened fasted conditions (Figure 3B). Gas production was not significantly affected upon the addition of *L. paracasei* DG compared with the blank control.

**Figure 4:**
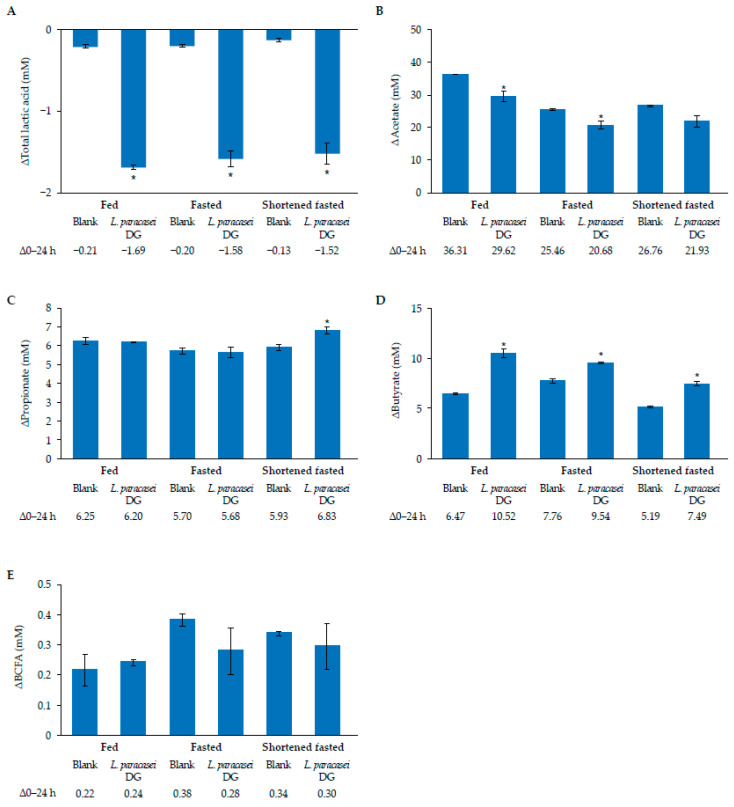
Microbial metabolic activity in short colonic incubations. Change (mean [SD]) in total lactic acid (**A**), acetate (**B**), propionate (**C**), butyrate (**D**), and BCFAs (**E**) upon colonic fermentation from 0 to 24 h in the presence of *L. paracasei* DG under fed, fasted, and shortened fasted conditions. *n* = 3 technical replicates. * Represents statistically significant differences compared with blank control for the respective condition (*p* < 0.05).

To assess microbial metabolic activity further, the changes in lactate and SCFA concentrations were quantified (Figure 4). Overall, the lactate concentration of the human inoculum during blank colonic control incubations decreased after 24 h from baseline (−0.21 to −0.13; Figure 4A). Addition of *L. paracasei* DG led to a larger reduction in lactate compared with blank control incubation across all conditions (−1.69 to −1.52 vs. −0.21 to −0.13; *p* < 0.05). The acetate concentration increased after 24 h during the blank incubation by 25.5–36.3 mM across all conditions (Figure 4B). Supplementation with *L. paracasei* DG resulted in a smaller increase compared with the blank control incubation under the fed condition (36.31 mM vs. 29.62 mM; *p* < 0.05) and fasted conditions (25.46 mM vs. 20.68 mM; *p* < 0.05). The propionate concentration increased from baseline after 24 h in the blank control incubation by 5.7–6.3 mM across the three conditions (Figure 4C). Supplementation with *L. paracasei* DG enhanced propionate production above the blank control incubations under shortened fasted conditions (6.83 mM vs. 5.93 mM; *p* < 0.05), but not for the other conditions. The butyrate concentration increased by 5.19–7.76 mM following 24 h of incubation from baseline in the blank control condition (Figure 4D). Supplementation with *L. paracasei* DG significantly enhanced the production of butyrate by 7.49–10.52 mM across all three conditions (*p* < 0.05) from baseline. No significant differences were observed in branched-chain fatty acid (BCFA) production (Figure 4E).

### 3.3. Quantification of L. paracasei DG Following 24 h of Colonic Incubation

**Figure 5:**
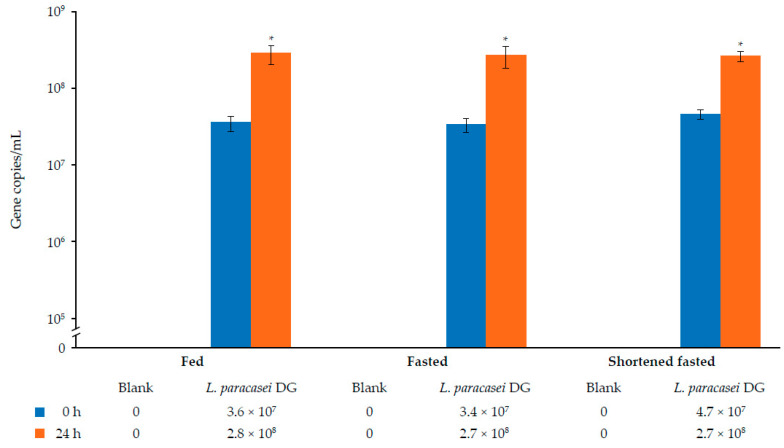
Quantitative PCR enumeration of *L. paracasei* DG following 24 h colonic incubation. Change (mean [SD]) of luminal *L. paracasei* DG (16S ribosomal RNA gene copies/mL) upon colonic incubation at baseline and 24 h in the presence of *L. paracasei* DG supplement compared with the blank control under fed, fasted, and shortened fasted conditions. *n* = 3 technical replicates. The total volume of the incubation was 700 mL. * Represents statistical differences between baseline and 24 h for the respective condition (*p* < 0.05).

Quantification of the 16S ribosomal RNA gene copies/mL in the blank control incubation showed no detectable *L. paracasei* DG gene copies in the donor sample at baseline and 24 h (Figure 5). Following incubation of the sample with *L. paracasei* DG supplementation, the gene copies/mL significantly increased across all three conditions (baseline vs. 24 h: 3.4–4.7 × 10^7^ copies/mL vs. 2.7–2.8 × 10^8^ copies/mL).

### 3.4. Composition of the Donor Microbial Community Following L. paracasei DG Incubation

Analysis of the donor microbial community through shallow shotgun sequencing demonstrated that similar microbial diversity was observed for all test conditions at the start of the incubation (Table 1). However, after 24 h of incubation, significantly higher microbial diversity was observed under fasted and shortened fasted conditions than under the fed condition (e.g., reciprocal Simpson’s index for the blank condition: fed, 6.0; fasted, 18.6; shortened fasted, 14.7). Administration of *L. paracasei* DG did not significantly affect microbial diversity compared with the blank control incubations under fed, fasted, and shortened fasted conditions, although a trend towards increased microbial diversity was observed under fed conditions (index: 7.2 vs. 6.0; not significant). An opposite trend was observed under fasted conditions (16.4 vs. 18.6; not significant).

The impact of supplementation with *L. paracasei* DG was assessed at the phylum (Supplementary Table S1), family (Supplementary Table S2), and species levels (Table 2; on the 25 most abundant andrelevant species in the samples). At baseline, the main bacterial phyla present in the microbial community of the selected donor were Actinobacteria, Bacteroidetes, and Firmicutes (Supplementary Table S1). Levels of Actinobacteria, Bacteroidetes, Firmicutes, Proteobacteria, and Verrucomicrobia increased after 24 h of incubation with the blank control compared with baseline. After 24 h of incubation with *L paracasei* DG, levels of Firmicutes phyla (fed condition), Proteobacteria (all conditions), and Verrucomicrobia (fed and shortened fasted conditions) were increased compared with those of the blank control (*p* < 0.05).

Several microbial communities were affected by *L. paracasei* DG supplementation under all tested conditions. Enterobacteriaceae levels (mainly attributable to *Escherichia coli*) were strongly reduced upon supplementation of *L. paracasei* DG under fed, fasted, and shortened fasted conditions compared with those of the blank control (Table 2; average relative abundance at 24 h: fed, 7.7% vs. 10.4%; fasted, 3.9% vs. 5.0%; shortened fasted, 4.5% vs. 6.0%). Furthermore, the abundance of *Blautia wexlerae* (a species of the Lachnospiraceae family) was consistently and significantly reduced upon treatment with *L. paracasei* DG compared with that of the blank control (average relative abundance at 24 h: fed, 1.3% vs. 1.6%; fasted, 1.2% vs. 1.9%; shortened fasted, 1.4% vs. 2.5%).

Under fed conditions, treatment with *L. paracasei* DG significantly increased the levels of several family members of the Firmicutes phyla (Clostridiaceae, Eubacteriaceae, Lachnospiraceae, and Lactobacillaceae; Supplementary Table S2) above those of the blank control following a 24 h incubation, mainly owing to the increased abundance of *Dorea longicatena* and *Agathobacter rectalis* (previously known as *Eubacterium rectale*) (Table 2). *L. paracasei* DG-induced changes in microbial composition under fasted and shortened fasted conditions were less pronounced than those for the blank control. Following 24 h of incubation with *L. paracasei* DG, levels of Bifidobacteriaceae (a member of the Actinobacteria phylum) were significantly higher than those in the blank control (average relative abundance under fasted conditions: 29.7% vs. 25.2%; *p* < 0.05; Supplementary Table S2). Similarly, Lactobacillaceae significantly increased following 24 h of incubation with *L. paracasei* DG under fasted conditions compared with that of the blank control (3.9% vs. 0.0%; *p* < 0.05). Levels of Clostridiales_u_f were significantly enhanced upon supplementation of *L. paracasei* DG under fasted conditions. Levels of Ruminococcaceae decreased upon supplementation of *L. paracasei* DG under both fasted and shortened fasted conditions (reaching significance under shortened fasted conditions; Supplementary Table S2). At a lower phylogenetic level, these changes were mainly attributed to decreased levels of *Ruminococcus* species upon supplementation with *L. paracasei* DG compared with those of the blank control (Table 2). However, levels of the butyrate-producing *Faecalibacterium prausnitzii* (also belonging to the Ruminococcaceae family) changed minimally.

## 4. Discussion

To provide mechanistic insight on the reported benefits observed for volunteers and patients receiving supplemental *L. paracasei* DG [9,10,11,26], this study investigated the survival and metabolic activity of *L. paracasei* DG capsules in a modified SHIME^®^ reactor. The results demonstrated that encapsulated *L. paracasei* DG survived conditions representative of the transition through the upper GI tract under fed, fasted, and shortened fasted conditions, resulting in the delivery of a sufficient dose of metabolically active cells into the target site of the colon. Orally administered probiotic microorganisms must survive through the harsh conditions of the upper GI tract at sufficient levels for delivery to the colon to have a probiotic effect [14]. Although engraftment was not formally tested in this study, the survival and activity of *L. paracasei* DG under simulated GI tract conditions, in combination with previous observations in patients, healthy adults, and children, suggest that colonic engraftment of *L. paracasei* DG is likely [5,6].

The main process occurring in the colon is microbial fermentation, which is due to the beneficial pH conditions and the constant provision to the gut flora of dietary non-digestible carbohydrates, host-derived glycans, and proteins [27,28]. In addition to measuring the metabolites directly, changes in the overall pH and the increased production of gases are useful indicators of microbial fermentation. Gas production is especially relevant in the patient context because rapid fermentation causes high initial gas production, which can potentially result in discomfort related to bloating and flatulence [29]. Supplementation of human faecal inoculum with *L. paracasei* DG reduced the pH, lactate production, and acetate production and increased the production of gases and butyrate without impacting propionate or BCFA levels. Butyrate is an essential metabolite used by colonocytes that supports the preservation of gut integrity and regulates immune and metabolic responses [30,31]. It is predominantly produced by the Firmicutes phylum and may protect against colorectal cancer [32]. Furthermore, increased butyrate levels were observed in patients with IBS receiving *L. paracasei* DG supplements who responded to treatment compared with pre-treatment levels [9]. The higher fermentative rate was linked to significantly higher levels of butyrate-producing Clostridiaceae, Eubacteriaceae, and Lachnospiraceae families.

Decreased lactate concentrations may appear initially counterintuitive because lactobacilli are one of the main producers of lactate [33]. However, these results suggest that *L. paracasei* DG may have colonized the microbial environment, altering microbial fermentation (specifically lactate and acetate consumption) via cross-feeding of other microbial species for the production of butyrate. Indeed, treatment with *L. paracasei* DG led to a significant reduction in the acetate-producing Enterobacteriaceae (under all conditions) and *Blautia wexlerae* (a Ruminococcus species; under fasted and shortened fasted conditions). Interestingly, as the Enterobacteriaceae family is linked with enteric diseases, the reduction of Enterobacteriaceae levels upon supplementation of *L. paracasei* DG may be beneficial. However, engraftment was not formally tested in this study.

Similar SHIME^®^ analyses with other *Lactobacillus* populations (*L. acidophilus, L. rhamnosus,* and *L. helveticus*), as single strains or in combination with other probiotics, have shown variable impacts on the production of SCFAs, including butyrate [34,35,36,37]. *L. acidophilus* results appeared the most similar to *L. paracasei* DG for the increased production of butyrate and expansion of Lactobacillus populations [34]. *L. paracasei* DG in combination with *Heyndrickxia coagulans* increased the proportion of butyrate-producing bacteria, despite a minimal detected increase in butyrate beyond ST end [20,36]. BCFAs are associated with significant negative health effects, and the absence of an increase after 24 h of incubation from baseline suggests that *L. paracasei* DG is not influencing proteolytic microbial activity, which could result in the release of toxic compounds [38]. In contrast, under fasted conditions, in certain formulations, *L. acidophilus* increased microbial BCFA concentrations after 24 h [20].

A key strength of this study was the use of the modified SHIME^®^ system, which allows for the investigation of microbial survival at different stages of the passage through the upper GI tract. Using the SHIME^®^ system allowed for investigation into the viability and metabolic activity of *L. paracasei* DG at each stage of digestion, providing key mechanistic insights into the survival of *L. paracasei* DG in adults and children receiving the probiotics. However, this study is limited to a single human faecal inoculum, as the experiments were conducted in 2020 and, due to the COVID-19 pandemic, it was difficult to recruit further donors safely. Moreover, given the limited number of replicates, direct assessment of distributional assumptions was not feasible. However, historical datasets generated under identical conditions have consistently shown residuals approximating a normal distribution. We therefore proceeded with parametric tests. The efficiency of quantitative PCR for *L. paracasei* DG was 89.3% and thus slightly below the recommended lower limit of 90%. The engraftment of *L. paracasei* DG within the colonic environment was also not formally tested. Further research is necessary to formally evaluate the engraftment of *L. paracasei* DG in the GI tract.

## 5. Conclusions

The viability and culturability of *L. paracasei* DG were minimally impacted by exposure to the low pH of the upper GI tract. Moreover, compared with the blank control, *L. paracasei* DG had a measurable effect on the metabolism of other microbiota following the 24 h colonic incubation. *L. paracasei* DG facilitated butyrate production and reduced the levels of acetate and lactate, indicating that *L. paracasei* DG stimulated cross-feeding interactions. These findings support the probiotic properties of *L. paracasei* DG and are in line with the previously demonstrated efficacy in healthy volunteers and patients with various GI diseases.

## Figures and Tables

**Table 1:** Reciprocal Simpson’s index.

	Fed		Fasted		Short Fasted	
	Blank	*L. paracasei* DG	Blank	*L. paracasei* DG	Blank	*L. paracasei* DG
0 h	25.7	26.2	24.7	24.3	26.6	26.8
24 h	6.0	7.2	18.6	16.4	14.7	14.4

The reciprocal Simpson’s index in colonic incubations at baseline (0 h) and after 24 h in the presence of encapsulated *L. paracasei* DG (*n* = 3) compared with blank control (*n* = 3) under fed, fasted, and shortened fasted conditions. The intensity of the green shading indicates the absolute diversity index, normalized for each time point, where the darker greens indicate a higher diversity index. None of the differences were statistically significant from the blank control incubation.

**Table 2:** Average relative abundance of the top 25 most abundant and relevant bacterial species at 0 h and 24 h after colonic incubation.

Phylum	Family	Species	0 h					
			Fed		Fasted		Shortened Fasted	
			Blank (%)	*L. paracasei* DG (%)	Blank (%)	*L. paracasei* DG (%)	Blank (%)	*L. paracasei* DG (%)
Actinobacteria	Bifidobacteriaceae	*Bifidobacterium adolescentis*	11.5	11.7	11.9	11.9	9.8	9.6
		*Bifidobacterium longum*	1.3	1.2	1.3	1.2	1.1	1.0
		*Bifidobacterium sp. 12_1_47BFAA*	1.5	1.4	1.4	0.9	1.3	0.9
	Coriobacteriaceae	*Collinsella aerofaciens*	2.4	2.4	2.8	2.7	2.1	2.1
		*Collinsella sp. 4_8_47FAA*	2.6	2.6	3.1	3.0	2.3	2.3
		*Senegalimassilia anaerobia*	0.3	0.3	0.3	0.3	0.3	0.3
Bacteroidetes	Bacteroidaceae	*Bacteroides coprocola*	5.9	6.1	6.9	7.2	8.0	7.4
		*Bacteroides dorei*	1.4	1.4	1.5	1.6	2.0	1.5
		*Bacteroides thetaiotaomicron*	0.4	0.4	0.5	0.5	0.5	**0.5**
		*Bacteroides vulgatus*	4.1	4.0	4.3	4.6	4.6	4.5
		*Bacteroides sp. 3_1_40A*	2.4	3.6	1.4	2.8	4.2	2.6
		*Bacteroides sp. 4_3_47FAA*	1.3	0.0	2.5	1.5	0.0	1.5
	Rikenellaceae	*Alistipes putredinis*	5.0	**4.5**	5.0	5.0	4.8	5.0
Firmicutes	Acidaminococcaceae	*Phascolarctobacterium succinatutens*	2.4	2.1	2.2	1.9	2.0	2.0
	Bacillaceae	*Bacillus clausii*	0.0	0.0	0.0	0.0	0.0	0.0
	Clostridiaceae	*Clostridium sp. L2-50*	4.3	4.2	4.0	3.4	4.2	4.2
	Lachnospiraceae	*Blautia wexlerae*	1.9	1.8	1.9	1.7	2.0	2.0
		*Dorea longicatena*	1.2	1.1	0.9	0.8	1.1	**1.0**
		*Agathobacter rectalis*	3.8	3.4	3.5	3.1	3.9	4.0
	Lactobacillaceae	*Lactobacillus paracasei*	0.0	**4.4**	0.0	**4.3**	0.0	**4.8**
	Ruminococcaceae	*Faecalibacterium prausnitzii*	5.8	5.2	5.6	5.3	4.7	4.8
		*Ruminococcus bromii*	6.0	5.2	5.9	5.5	6.1	**5.4**
		*Ruminococcus sp. 5_1_39BFAA*	2.2	2.1	2.2	2.0	2.4	2.3
Proteobacteria	Enterobacteriaceae	*Escherichia coli*	0.0	0.1	0.0	0.0	0.1	0.1
	Sutterellaceae	*Sutterella wadsworthensis*	0.9	0.8	0.5	0.6	0.8	0.8
			**24 h**					
Actinobacteria	Bifidobacteriaceae	*Bifidobacterium adolescentis*	38.8	35.3	19.5	21.6	23.2	23.7
		*Bifidobacterium longum*	2.9	2.8	2.0	**2.5**	2.4	2.5
		*Bifidobacterium sp. 12_1_47BFAA*	3.3	3.3	1.5	2.8	2.7	2.9
	Coriobacteriaceae	*Collinsella aerofaciens*	1.0	1.0	2.7	2.3	1.6	**1.3**
		*Collinsella sp. 4_8_47FAA*	1.2	1.2	3.0	2.6	1.8	**1.5**
		*Senegalimassilia anaerobia*	2.7	**2.2**	3.6	3.2	3.3	2.8
Bacteroidetes	Bacteroidaceae	*Bacteroides coprocola*	0.0	0.0	0.1	0.1	0.1	0.1
		*Bacteroides dorei*	1.0	1.0	1.6	1.4	1.7	1.6
		*Bacteroides thetaiotaomicron*	1.5	1.3	2.8	2.4	2.2	1.8
		*Bacteroides vulgatus*	2.4	2.3	3.0	3.5	3.4	3.6
		*Bacteroides sp. 3_1_40A*	0.0	0.0	0.0	0.0	0.0	0.0
		*Bacteroides sp. 4_3_47FAA*	2.2	2.1	2.8	3.1	3.1	3.3
	Rikenellaceae	*Alistipes putredinis*	0.7	0.6	1.2	1.2	1.4	1.6
Firmicutes	Acidaminococcaceae	*Phascolarctobacterium succinatutens*	0.8	0.7	1.2	1.0	1.4	**1.2**
	Bacillaceae	*Bacillus clausii*	0.0	0.0	0.0	0.0	0.0	0.0
	Clostridiaceae	*Clostridium sp. L2-50*	0.4	0.0	0.4	0.0	0.0	0.0
	Lachnospiraceae	*Blautia wexlerae*	1.6	**1.3**	1.9	**1.2**	2.5	**1.4**
		*Dorea longicatena*	0.7	**1.0**	2.8	2.7	1.0	0.9
		*Agathobacter rectalis*	0.6	**0.8**	1.9	1.6	1.2	1.5
	Lactobacillaceae	*Lactobacillus paracasei*	0.0	**3.7**	0.0	**3.6**	0.0	**4.5**
	Ruminococcaceae	*Faecalibacterium prausnitzii*	0.8	1.0	1.4	1.3	1.9	1.8
		*Ruminococcus bromii*	1.6	1.7	3.0	2.3	3.2	2.6
		*Ruminococcus sp. 5_1_39BFAA*	1.9	1.6	2.3	**1.5**	3.0	**1.7**
Proteobacteria	Enterobacteriaceae	*Escherichia coli*	10.4	**7.7**	5.0	**3.9**	6.0	**4.5**
	Sutterellaceae	*Sutterella wadsworthensis*	1.6	1.9	3.0	2.4	1.0	1.2

Average relative abundance of the top 25 bacterial species in colonic incubation at baseline (0 h) and after 24 h in the presence of encapsulated *L. paracasei* DG (*n* = 3) compared with blank (*n* = 3) under fed, fasted, and shortened fasted conditions as a proportion and coloured from white to green to indicate the level of enrichment. The intensity of the shading indicates the proportion, normalized for each time point, where the darker greens indicate a higher proportion of the bacterial species. Statistically significant differences from the blank incubation are indicated in bold (*p* < 0.05).

## Data Availability

The data presented in this study are available on request from the corresponding author due to the proprietary nature of the methods used.

## Supplementary Materials

The following supporting information can be downloaded at https://www.mdpi.com/article/10.3390/nu17182952/s1. Supplementary Table S1. Average relative abundance of different phyla at 0 h and 24 h after colonic incubation. Supplementary Table S2. Average relative abundance of different microbial families at 0 h and 24 h after colonic incubation. Supplementary Figure S1. SHIME^®^ experimental model. Supplementary Figure S2. Efficiency of quantitative PCR for *L. paracasei* DG.

## Abbreviations

The following abbreviations are used in this manuscript:

BCFA	Branched-chain fatty acid
CFU	Colony-forming units
DUO	Duodenum
GI	Gastrointestinal
ILE	Ileum
IBS	Irritable bowel syndrome
JEJ	Jejunum
PCR	Polymerase chain reaction
SCFA	Short-chain fatty acid
SHIME	Simulator of the Human Intestinal Microbial Ecosystem
SD	Standard deviation
Sp.	Species
ST	Stomach
SUDD	Symptomatic uncomplicated diverticular disease
u_f	unclassified family
