# Supplementary material for: Survival and Impact on Microbial Diversity of Lacticaseibacillus paracasei DG in a Simulation of Human Intestinal Microbial Ecosystem
Source: Nutrients. 2025 Sep 13;17(18):2952. doi: 10.3390/nu17182952 (PMC12472629; doi:10.3390/nu17182952)
Supplement: Supplementary file 1 [file nutrients-17-02952-s001.zip › nutrients-3822632-supplementary.pdf]

Supplementary Table S1. Average relative abundance of different phyla at 0 h and 24 h after colonic incubation.

Phylum	0 h					
	Fed		Fasted		Shortened Fasted	
	Blank (%)	*L. paracasei* DG (%)	Blank (%)	*L. paracasei* DG (%)	Blank (%)	*L. paracasei* DG (%)
Actinobacteria	21.1	21.1	22.4	21.1	17.9	17.3
Bacteroidetes	32.3	31.5	34.5	36.0	37.0	34.7
Firmicutes	44.7	45.6	41.6	41.4	43.2	**46.4**
Proteobacteria	1.4	1.3	1.0	1.0	1.4	1.2
Verrucomicrobia	0.5	0.5	0.5	0.5	0.4	0.4
	**24 h**					
Actinobacteria	54.8	50.2	35.1	**38.5**	38.7	38.7
Bacteroidetes	14.7	14.5	24.0	22.3	22.6	22.0
Firmicutes	16.8	**24.2**	31.6	31.6	30.6	32.3
Proteobacteria	13.3	**10.8**	8.8	**7.4**	7.7	**6.7**
Verrucomicrobia	0.4	**0.3**	0.4	0.3	0.4	**0.3**

The abundance of different phyla in colonic incubation at baseline (0 h) and after 24 h in the presence of encapsulated *L. paracasei* DG ($n = 3$) compared with a blank control ($n = 3$) under fed, fasted, and shortened fasted conditions as a proportion and coloured from white to green to indicate the level of enrichment. The intensity of the shading indicates the proportion, normalized for each time point, where the darker greens indicate a higher proportion of the bacterial species. Statistically significant differences from the blank incubation are indicated in bold ($p < 0.05$).

Supplementary Table S2. Average relative abundance of different microbial families at 0 h and 24 h after colonic incubation.

Phylum	Family	0 h					
		Fed		Fasted		Shortened Fasted	
		Blank (%)	*L. paracasei* DG (%)	Blank (%)	*L. paracasei* DG (%)	Blank (%)	*L. paracasei* DG (%)
Actinobacteria	Atopobiaceae	0.0	0.1	0.1	0.0	0.1	0.0
	Bifidobacteriaceae	15.3	15.4	15.6	14.7	12.9	12.2
	Coriobacteriaceae	5.4	5.4	6.3	6.0	4.7	4.8
	Eggerthellaceae	0.1	0.1	0.1	0.1	0.1	0.1
	Propionibacteriaceae	0.2	0.2	0.2	0.2	0.2	0.2
Bacteroidetes	Bacteroidaceae	19.9	19.6	21.6	23.0	24.1	22.5
	Bacteroidales_u_f	0.0	0.0	0.1	0.1	0.1	0.0
	Barnesiellaceae	0.1	0.1	0.1	0.1	0.1	0.1
	Odoribacteraceae	0.7	0.8	0.9	0.9	1.0	0.9
	Porphyromona-daceae	0.4	0.4	0.5	0.4	0.5	0.4
	Prevotellaceae	1.3	1.2	1.1	1.2	1.2	1.0
	Rikenellaceae	8.5	7.8	8.6	8.7	8.3	8.4
	Tannerellaceae	1.4	1.5	1.6	1.5	1.7	**1.3**
Firmicutes	Acidaminococcaceae	2.4	2.1	2.2	1.9	2.0	2.0
	Bacillaceae	0.0	0.0	0.0	0.0	0.0	0.0
	Christensenellaceae	0.2	**0.2**	0.2	0.2	0.2	**0.2**
	Clostridiaceae	4.5	4.4	4.3	3.7	4.4	4.5
	Clostridiales_u_f	0.4	0.4	0.3	0.6	0.4	0.6
	Enterococcaceae	0.0	**0.1**	0.0	0.0	0.0	0.0
	Erysipelotrichaceae	1.1	1.0	1.0	0.9	1.0	1.0
	Eubacteriaceae	2.2	2.0	1.9	1.6	2.3	**1.8**
	Lachnospiraceae	14.9	13.8	13.3	11.3	15.2	14.4
	Lactobacillaceae	0.1	**4.7**	0.0	**4.6**	0.1	**5.2**
	Oscillospiraceae	1.7	1.4	1.7	1.4	1.5	1.5

	Peptostreptococcaceae	0.1	0.1	0.1	0.1	0.1	0.1
	Ruminococcaceae	16.9	15.2	16.5	15.1	15.9	15.0
	Streptococcaceae	0.1	0.1	0.1	0.1	0.1	0.1
Proteobacteria	Burkholderiales_u_f	0.0	0.1	0.0	0.0	0.0	0.0
	Desulfovibrionaceae	0.3	0.3	0.5	0.4	0.4	0.3
	Enterobacteriaceae	0.0	0.1	0.0	0.0	0.1	0.1
	Oxalobacteraceae	0.0	0.0	0.0	0.0	0.1	**0.0**
	Sutterellaceae	0.9	0.8	0.5	0.6	0.8	0.8
Verrucomicrobia	Akkermansiaceae	0.5	0.5	0.5	0.5	0.4	0.4
		**24 h**					
Actinobacteria	Atopobiaceae	0.0	0.0	0.0	0.0	0.0	0.0
	Bifidobacteriaceae	49.5	45.3	25.2	**29.7**	31.4	32.3
	Coriobacteriaceae	5.2	4.6	9.5	8.3	6.9	6.0
	Eggerthellaceae	0.1	0.2	0.4	0.4	0.3	0.3
Bacteroidetes	Bacteroidaceae	9.3	8.8	15.9	14.6	14.8	14.1
	Bacteroidales_u_f	0.3	0.2	0.0	0.2	0.0	0.0
	Odoribacteraceae	0.0	0.1	0.1	0.1	0.2	0.2
	Porphyromonadaceae	0.5	**0.8**	1.2	1.1	0.8	0.8
	Prevotellaceae	0.0	0.0	0.1	0.0	0.0	0.0
	Rikenellaceae	2.5	2.3	3.4	3.4	3.6	4.0
	Tannerellaceae	2.0	2.3	3.4	**2.8**	3.2	2.8
Firmicutes	Acidaminococcaceae	0.8	0.7	1.2	1.0	1.4	**1.2**
	Bacillaceae	0.0	0.0	0.0	0.0	0.0	0.0
	Christensenellaceae	0.0	0.1	0.3	0.1	0.1	0.1
	Clostridiaceae	0.5	**0.7**	0.7	0.6	1.2	1.0
	Clostridiales_u_f	0.3	0.3	1.2	**1.7**	1.0	1.0
	Enterococcaceae	0.1	0.0	0.1	**0.0**	0.1	**0.0**
	Erysipelotrichaceae	0.5	0.5	1.9	1.6	0.9	0.6

	Eubacteriaceae	1.2	**1.8**	1.1	1.1	0.9	0.9
	Lachnospiraceae	8.3	**11.0**	16.1	14.4	15.7	15.1
	Lactobacillaceae	0.1	**4.0**	0.0	**3.9**	0.0	**4.9**
	Oscillospiraceae	0.2	0.2	1.0	0.9	0.4	0.4
	Ruminococcaceae	4.7	4.8	7.8	6.2	8.9	**7.0**
	Streptococcaceae	0.0	0.0	0.2	0.1	0.1	0.1
Proteobacteria	Desulfovibrionaceae	1.2	1.1	0.8	0.9	0.7	**1.0**
	Enterobacteriaceae	10.5	**7.8**	5.0	**3.9**	6.0	**4.5**
	Moraxellaceae	0.1	0.1	0.0	0.1	0.0	0.0
	Sutterellaceae	1.6	1.9	3.0	2.4	1.0	1.2
Verrucomicrobia	Akkermansiaceae	0.4	**0.3**	0.4	0.3	0.4	**0.3**

The abundance of different phyla in colonic incubation at baseline (0 h) and after 24 h in the presence of encapsulated *L. paracasei* DG ($n = 3$) compared with a blank control ($n = 3$) under fed, fasted, and shortened fasted conditions as a proportion and coloured from white to green to indicate the level of enrichment. The intensity of the shading indicates the proportion, normalized for each time point, where the darker greens indicate a higher proportion of the bacterial species. Statistically significant differences from the blank incubation are indicated in bold ($p < 0.05$).

Supplementary Figures

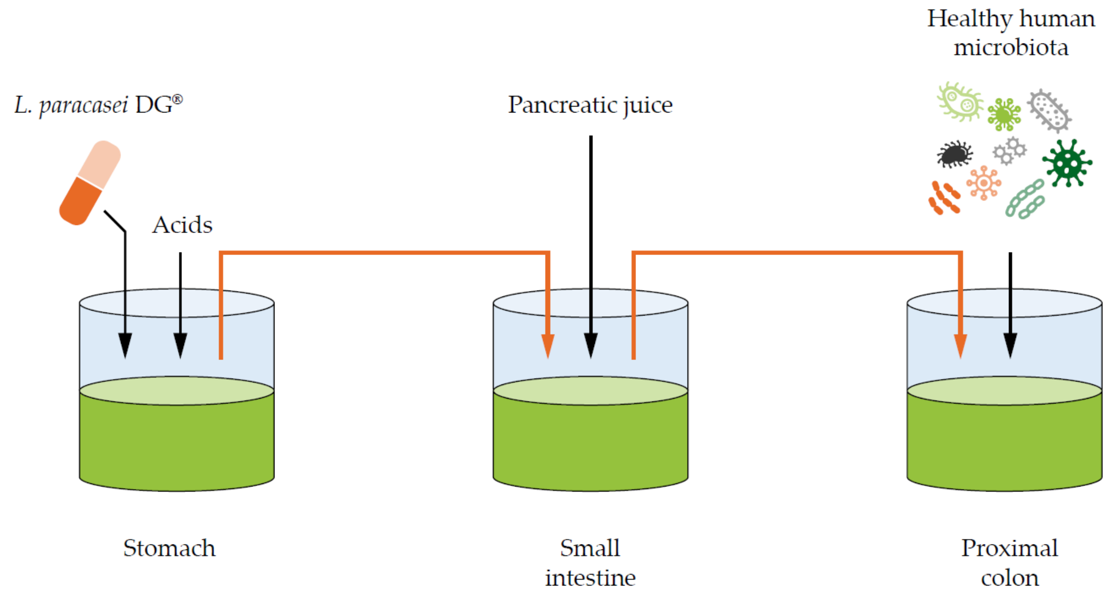

Supplementary Figure S1. SHIME® experimental model. SHIME® model set-up consisting of three reactors in a series resembling the GI tract under fed, fasted, and shortened fasted conditions. GI, gastrointestinal; SHIME®, Simulator of the Human Intestinal Microbial Ecosystem.

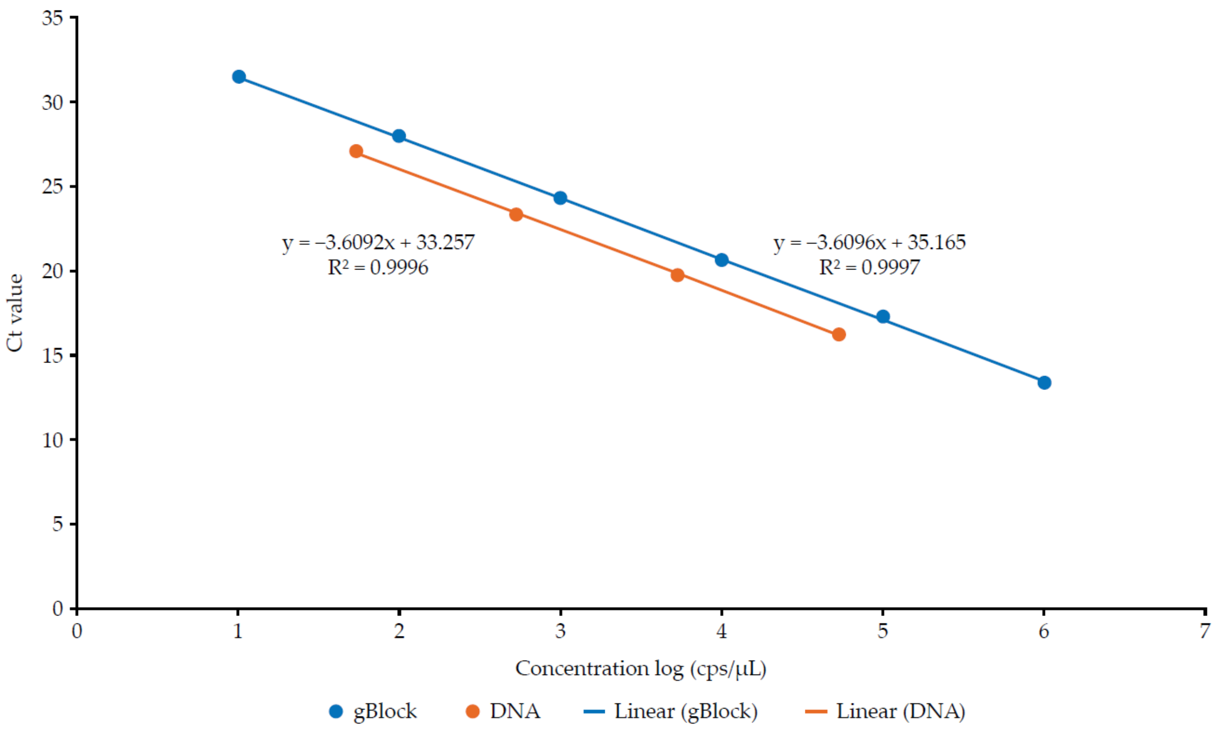

Supplementary Figure S2. Efficiency of quantitative PCR for *L. paracasei* DG. Ct values are plotted against the log concentration of *L. paracasei* DG in the test product (DNA; orange) and against the actual calibration curve for quantification of *L. paracasei* DG by quantitative PCR obtained through plotting Ct values against their corresponding concentrations in the gBlocks gene fragment (blue). Linear models were applied. The amplification efficiency was 89.3%. Cps, copies; Ct, cycle threshold; PCR, polymerase chain reaction
